# Matrix metalloprotease-1 inhibits and disrupts *Enterococcus faecalis* biofilms

**DOI:** 10.1371/journal.pone.0210218

**Published:** 2019-01-11

**Authors:** Lokender Kumar, Christopher R. Cox, Susanta K. Sarkar

**Affiliations:** 1 Department of Physics, Colorado School of Mines, CO, United States of America; 2 Department of Chemistry, Colorado School of Mines, CO, United States of America; University of Georgia, UNITED STATES

## Abstract

*Enterococcus faecalis* is a major opportunistic pathogen that readily forms protective biofilms leading to chronic infections. Biofilms protect bacteria from detergent solutions, antimicrobial agents, environmental stress, and effectively make bacteria 10 to 1000-fold more resistant to antibiotic treatment. Extracellular proteins and polysaccharides are primary components of biofilms and play a key role in cell survival, microbial persistence, cellular interaction, and maturation of *E*. *faecalis* biofilms. Degradation of biofilm components by mammalian proteases is an effective antibiofilm strategy because proteases are known to degrade bacterial proteins leading to bacterial cell lysis and growth inhibition. Here, we show that human matrix metalloprotease-1 inhibits and disrupts *E*. *faecalis* biofilms. MMPs are cell-secreted zinc- and calcium-dependent proteases that degrade and regulate various structural components of the extracellular matrix. Human MMP1 is known to degrade type-1 collagen and can also cleave a wide range of substrates. We found that recombinant human MMP1 significantly inhibited and disrupted biofilms of vancomycin sensitive and vancomycin resistant *E*. *faecalis* strains. The mechanism of antibiofilm activity is speculated to be linked with bacterial growth inhibition and degradation of biofilm matrix proteins by MMP1. These findings suggest that human MMP1 can potentially be used as a potent antibiofilm agent against *E*. *faecalis* biofilms.

## Introduction

The Centers for Disease Control and Prevention (CDC) estimates that there are at least 2 million antibiotic-resistant infections annually in the U.S. resulting in around 23,000 deaths [1]. Bacteria develop resistance to antimicrobial agents by evolving molecular mechanisms including targeted mutations, efflux pumps, and enzyme modifications [2]. Bacteria that are not innately resistant to antibiotics can also become resistant by forming persistent biofilms that lead to chronic infections [3]. The National Institute of Health reports that 80% of total human bacterial infections are biofilm-associated [4]. Biofilms are surface-associated, three dimensional bacterial communities surrounded by an extracellular matrix [5] that protect cells from antibiotics and immune cell attack [6, 7]. Biofilm matrices act as physical barriers to antibiotics and create a favorable ecological niche for long-term survival under harsh environmental and nutrient-poor conditions [8]. As such, biofilm-associated infections can become highly resistant to antibiotic therapy [9].

*Enterococcus faecalis* is a common Gram-positive etiologic agent of nosocomial and community acquired infections of burn and surgical wounds, urinary tract, abdominal, pelvic, gut, and endocarditis [8, 10–12] with high rates of morbidity and mortality [13]. *E*. *faecalis* attach efficiently to biotic and abiotic surfaces and secrete a protective extracellular matrix leading to formation of multi-layered antibiotic resistant biofilms [14]. In this context, enzymatic degradation of biofilms is believed to be an effective anti-biofilm strategy [15]. Alpha-amylase, bromelain, and papain have been found to significantly inhibit *S*. *aureus* biofilm formation [16]. Donelli *et al*. showed that β-N-acetylglucosaminidase purified from *Actinobacillus actinomycetemcomitans* exerted hydrolytic activity against exopolysaccharide (EPS) matrix and sensitized *staphylococcus* biofilms to antimicrobial agents [17]. In another study, co-administration of alginate-degrading enzyme alginate lyase and ceftazidime degraded EPS resulting in disruption of *Pseudomonas aeruginosa* biofilms [18]. Proteases are known to degrade membrane proteins (adhesins) and matrix proteins, which are responsible of initial attachment of cells to solid surfaces and adjacent bacterial cells [19, 20]. Bacterial cell signaling is regulated during biofilm formation by secretion of autoinducer peptides. Disruption of these signaling peptides using non-specific proteases is another potentially effective anti-biofilm approach [21]. For example, trypsin is a mammalian broad-spectrum protease that cleaves peptide bonds between C-terminal lysine or arginine and inhibits biofilm formation by *Pseudomonas aeruginosa*, *Streptococcus mitis*, *Actinomyces radicidentis* and *Staphylococcus epidermidis* [22–24]. Proteinase K, is another broad spectrum mammalian serine protease that exhibits broad-spectrum protease activity. This enzyme has been shown to inhibit biofilm formation against a range of Gram-negative and Gram-positive bacteria, including *Staphylococcus heamolyticus*, *Staphylococcus aureus*, *Staphylococcus lugdunensis and Escherichia coli* [22, 25–27]. Serratopeptidase from *Serratia marcescens* in combination with ofloxacin has a strong inhibiting effect against *P*. *aeruginosa* and *S*. *epidermidis* biofilms [28]. Recently, ficin, a nonspecific protease was reported to significantly inhibit *S*. *aureus* biofilm formation and enhance the efficacy of conventional antibiotics by disruption of biofilm matrix [29].

Human matrix metalloproteases (MMPs) are essential for tissue remodeling and can degrade a wide range of matrix and non-matrix associated proteins [30]. In particular, MMP1, a collagenase that is known to degrade type-1 collagen, can also degrade various structural components of the extracellular matrix (ECM) [31]. MMP1 has also been shown to play a role in the immune response to HIV, Hepatitis B, *Helicobacter pylori*, and *Mycobacterium tuberculosis* [32] and in inflammation [33]. Motivated by these broad-spectrum protease activities of MMP1, we investigated anti-biofilm effect of MMP1 against *E*. *Faecalis* biofilms. Here we report that MMP1 significantly inhibits and disrupts *E*. *faecalis* biofilms.

## Results

### Crystal violet (CV) assay to quantify inhibitory effect of MMP1 on biofilms

**Fig 1:**
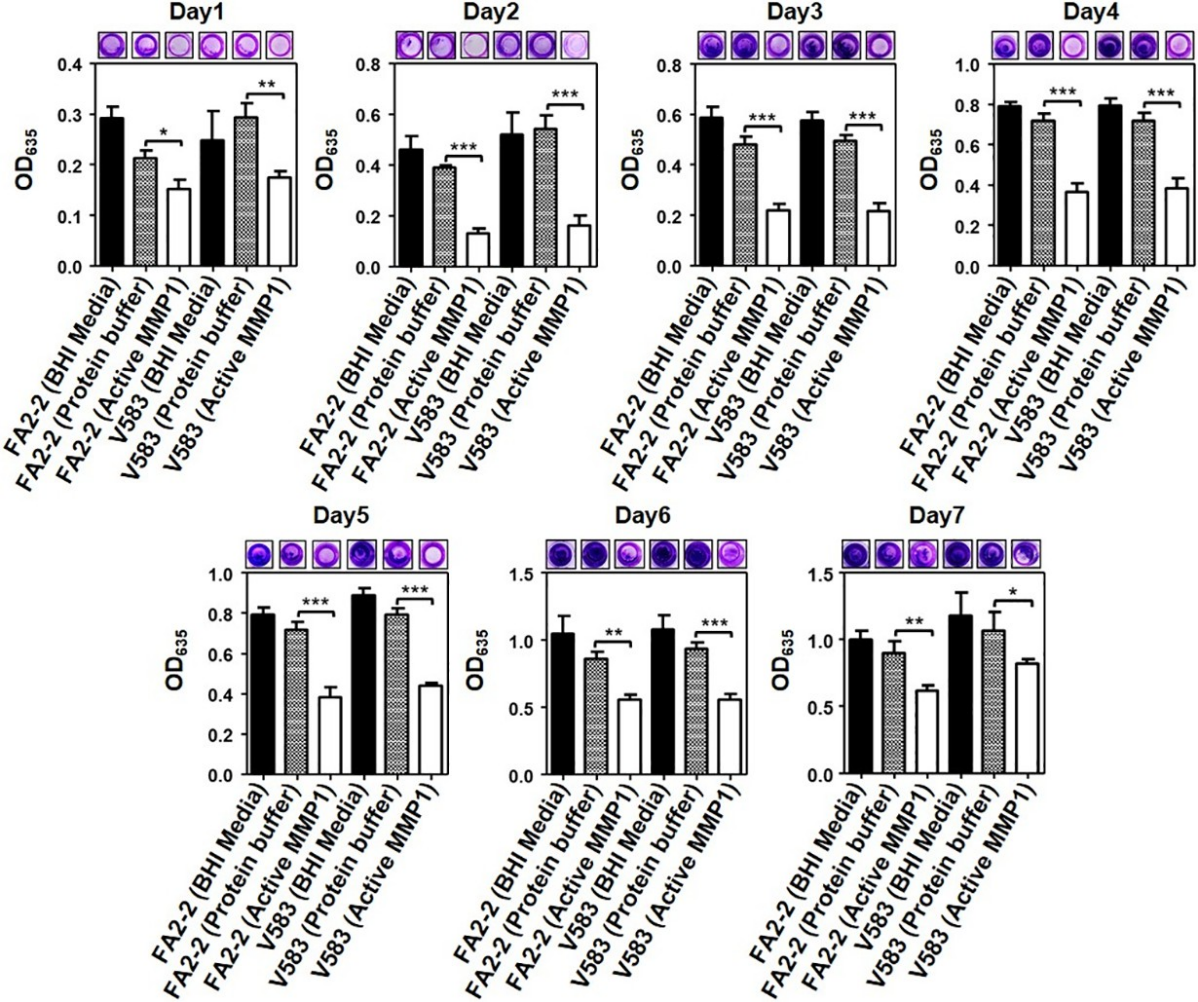
Inhibitory effect of MMP1 on *E*. *faecalis* biofilms. Absorption of CV stain at 635 nm (OD_635_) of MMP1-treated biofilms of *E*. *faecalis* FA2-2 and V583 strains over seven days. Experiments with BHI media and protein buffer were used as controls. For inhibition experiments, biofilms were grown in presence of MMP1 from day0 to day7. Typical images of wells in microtiter plates are given at the top of each panel. * indicates p-value: *<0.01, **<0.01, and ***<0.001. Error bars on the data points represent the standard deviations of 3 technical repeats.

To quantify the biofilm inhibition effect, biofilms were grown in the presence of MMP1. The inhibition effect of MMP1 on biofilm formation was quantified by CV staining [34] (see Methods for detailed procedures). To determine the baseline activity of CV assay, experiments with only BHI media were performed. **Fig 1** shows the results of seven day biofilm growth by FA2-2 (vancomycin susceptible) and V583 (vancomycin resistant). The images of wells captured by a camera clearly showed the inhibition effect even for 7-day-old inhibition experiment (**S1 Fig**).The use of Triton X-100 in the protein buffer had a small effect on the biofilms in some cases; however, in all cases the effect of protein buffer plus MMP1 showed more significant effect on the biofilm formation for both strains. Before dissolving CV stain in acetic acid, imaging of wells indicated visible reduction in bacterial biomass after MMP1 treatment as a qualitative indicator of inhibition.

### CV assay to quantify disruption effect of MMP1 on biofilms

**Fig 2:**
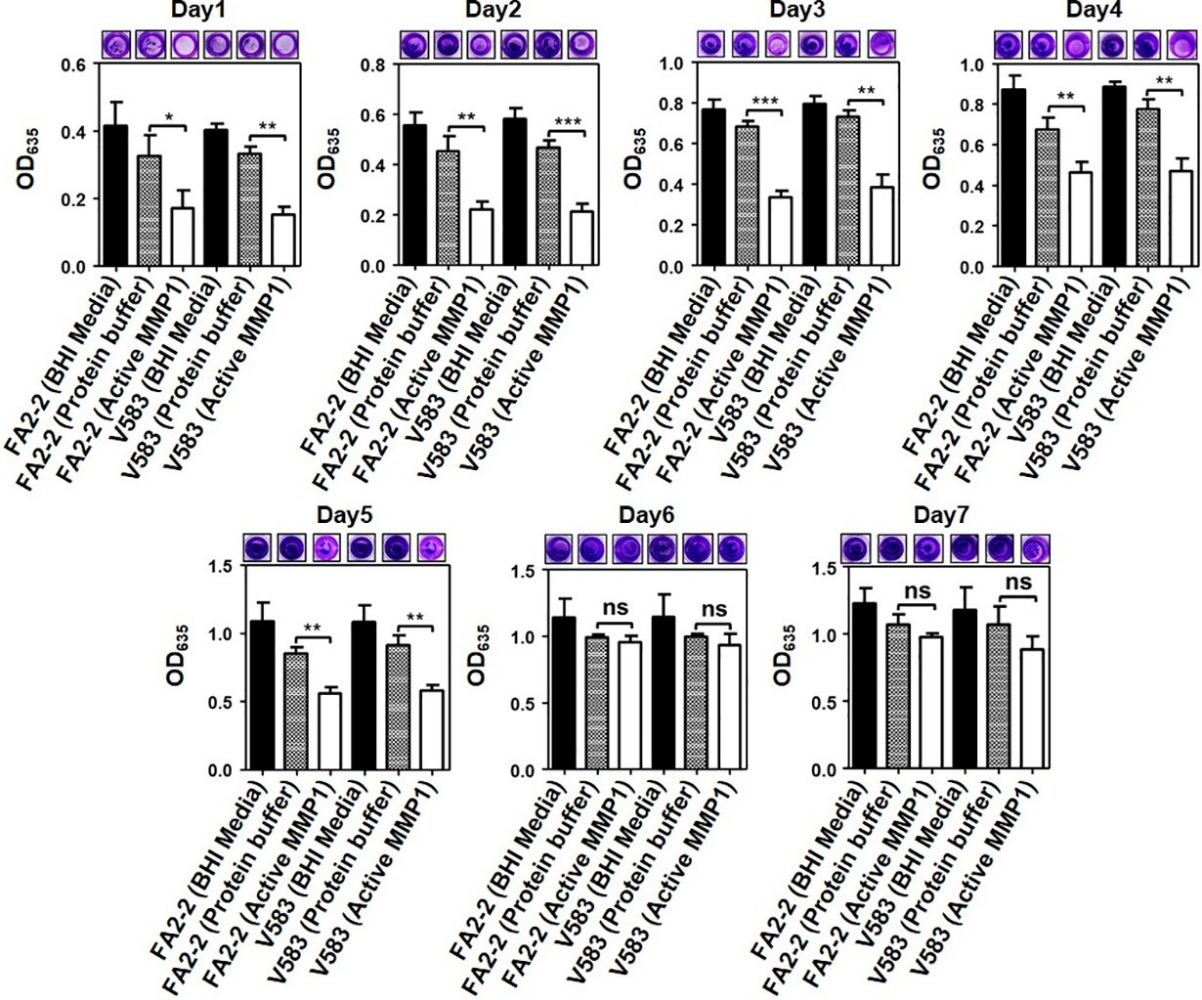
Disruptive effect of MMP1 on *E*. *faecalis* biofilms. Absorption of CV stain at 635 nm (OD_635_) of MMP1-treated biofilms of FA2-2 and V583 strains over seven days. Experiments with BHI media and protein buffer were used as controls. For disruption experiments, biofilms were first grown in BHI media without MMP1 for desired duration, followed by MMP1 treatment. Typical images of wells in microtiter plates are given at the top of each panel. * indicates p-value: *<0.01, **<0.01, and ***<0.001; ns indicates that the effect is not significant. Error bars on the data points represent the standard deviations of 3 technical repeats.

To quantify the biofilm disruption effect, biofilms were first grown for the desired duration in the absence of MMP1 and then treated with MMP1. It is likely that biofilm forming pathogens would already form a biofilm by the time of an accurate clinical diagnosis. Therefore, an ideal antibiofilm agent should also be able to effectively disrupt the established biofilms. Hence, we quantified the effect of MMP1 on established biofilms of *E*. *faecalis* strains (see Methods for detailed procedures). MMP1 effectively disrupted 1-day-old to 5-day-old biofilms (**Fig 2**). However, 6-day-old and 7-day-old biofilms were not completely disrupted by MMP1. Results of quantitative CV assay was qualitatively confirmed by images of wells stained with crystal violet.

### Scanning electron microscope (SEM) to image biofilm architecture of established biofilms

**Fig 3:**
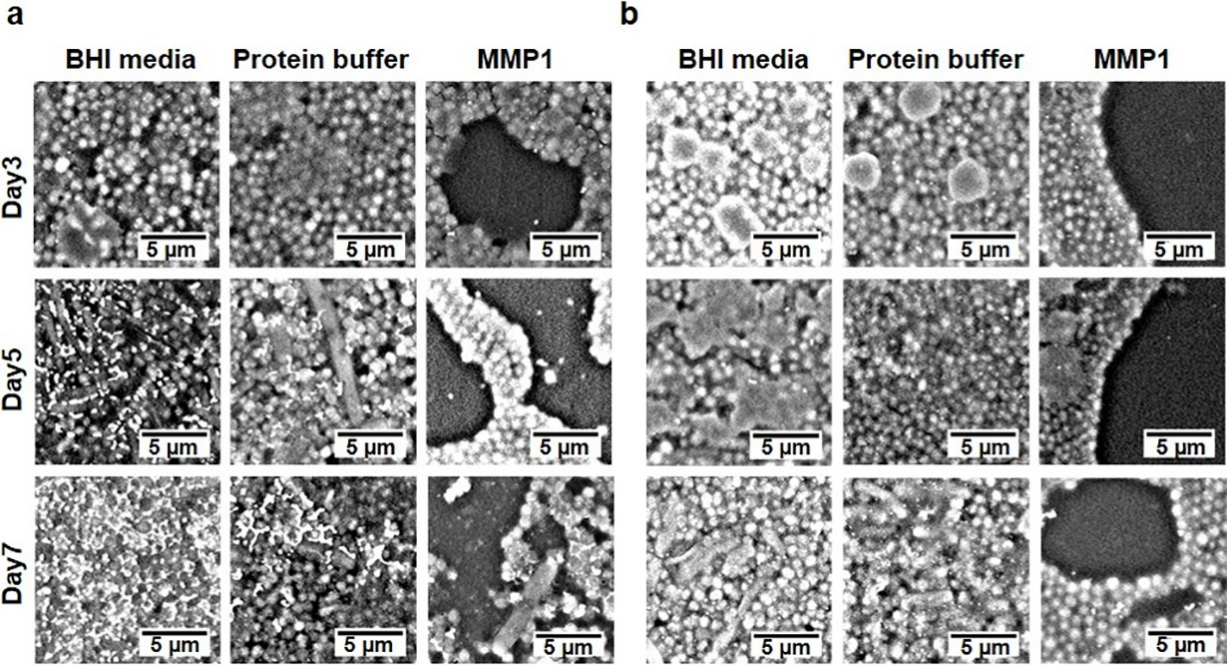
SEM micrographs of established *E*. *faecalis* biofilms. Biofilms were first grown for 3 to 7 days and then treated with MMP1. (**a**) Vancomycin susceptible strain FA2-2 and (**b**) Vancomycin resistant strain V583. In comparison to the control experiments, active MMP1 led to disruption of biofilms resulting in more empty spaces without any bacteria.

Observation of inhibition and disruption using CV assay (**Figs 1** and **2**) clearly indicated that MMP1 inhibited and disrupted *E*. *faecalis* biofilms. To investigate changes in biofilm architecture after treatment with MMP1 for 24 hr, we grew biofilms on plastic coverslips and imaged using environmental SEM without extra sample preparation and high vacuum necessary for conventional electron microscopy (see Methods for detailed procedures). This approach allowed for observation of changes in the extracellular polymeric substances (EPS) caused by sample processing even though we achieved lower resolution compared to the conventional electron microscopy. Further, we tested the effect of MMP1 on 3-day-old to 7-day-old established biofilms. MMP1-treated biofilms showed less bacterial colonization and high disruption in biofilm architecture as compared to the control biofilms (**Fig 3A** and **3B**; **S2** and **S3 Figs**). SEM images of 3-day-old biofilms without MMP1 treatment showed uniform layers of biofilm, whereas MMP1-treated biofilms showed a sporadic layer with patches of cells and large areas of clearance. Similar disruption of 5-day-old biofilms were observed after MMP1 treatment. For 7-day-old biofilms, MMP1 treatment did not completely destabilized the biofilm and thin layers of cells with lesser amounts of observable biofilm matrix were observed. It should be noted that the extended structures in SEM images (**Fig 3A** and **3B**) were not due to bacterial contamination because controls with only media did not lead to any growth over the course of experiments and similar structural features have been reported before for *E*. *faecalis* biofilms [35, 36].

### Confocal laser scanning microscopy to check viability of *E. faecalis* cells in the structural context of biofilms grown in the presence of MMP1

**Fig 4:**
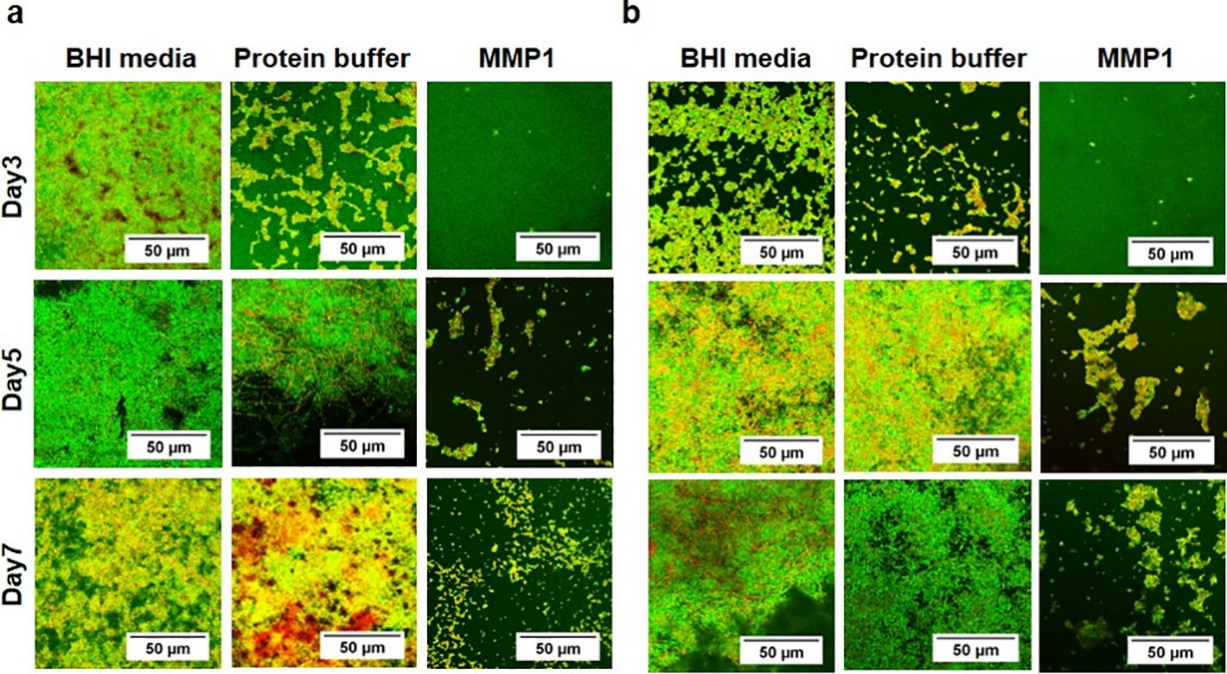
Confocal microscopy of *E*. *faecalis* biofilms. Biofilms were grown for 3 to 7 days in the presence of MMP1. (**a**) Vancomycin susceptible strain FA2-2 and (**b**) Vancomycin resistant strain V583. Orange and green areas indicate the presence of live and dead bacteria respectively. The amount of live and dead bacteria did not show consistent pattern because of the compounding effects of natural cell death, Triton X100, MMP1, and sample preparation.

Neither CV assays nor SEM informed whether MMP1 has an impact on bacterial viability. To perform live/dead assays and check the viability of bacteria, we grew biofilms on plastic coverslips in the presence of MMP1 and imaged biofilms by CLSM after staining with acridine orange and propidium iodide (see Methods for detailed procedures). Cells with compromised membranes stained red/orange, whereas viable bacteria with intact cell structure stained green. Confocal laser scanning microscopy showed the presence of both live and dead bacteria in varying amounts in 3-day-old to 7-day-old biofilms (**Fig 4A** and **4B**). For 3-day-old biofilms, we observed more viable bacterial cells than dead cells. For older biofilms, less viable cells were observed for both MMP1-treated and control biofilms as expected. No consistent pattern of live/dead cells was observed for different experimental conditions arising due to the compounding effects of deaths caused due to natural life cycle, Triton X100 in protein buffer, potential antibacterial effect of MMP1 and sample preparation for confocal imaging. Additionally, MMP1-treated biofilms had less biomass because cells were not able to attach and form biofilms. It should be noted that the control with protein buffer containing Triton X-100 had a measurable effect especially at day 3, which disappeared at later stages. Triton X-100 is a known antimicrobial detergent against Gram-positive and Gram-negative bacteria [37, 38] and lyse the bacterial cells by targeting bacterial membranes. Therefore, Triton X-100 affects thinner biofilms at the early stage. However, older biofilms become thick enough to prevent Triton X-100 from penetrating the biofilms and killing the resident bacteria. Triton X-100 is also known to stabilize proteins and we have used Triton X-100 in MMP1 purification buffer. Therefore, experiments with protein buffer containing Triton X-100 is an important control.

### Colony forming unit (CFU) assay to quantify viable cells in inhibition and disruption of biofilms by MMP1

**Fig 5:**
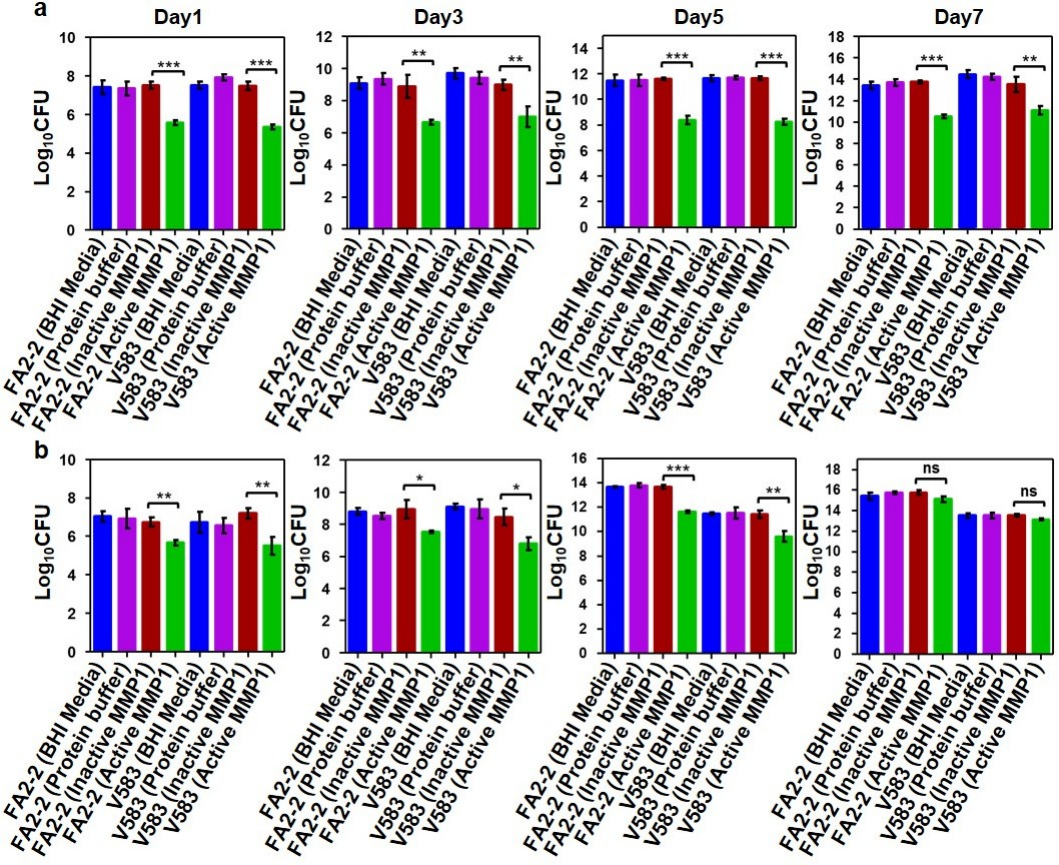
Colony forming unit (CFU) assay to quantify viable cells in *E*. *faecalis* biofilms. Quantification (Log _10_ CFU) of live bacterial cells in 1, 3 and 7 day old *E*. *faecalis* FA2-2 and V583 biofilms treated with and without MMP1 under inhibition (**a**) and disruption (**b**) conditions. For inhibition experiments, biofilms were grown in presence of MMP1 from day0 to day7. For disruption experiments, biofilms were first grown in BHI media without MMP1 for desired duration, followed by MMP1 treatment. * indicates p-value: *p<0.01, **p<0.01 and ***p<0.001; ns indicates that the effect is not significant. Error bars on the data points represent the standard deviations of 3 technical repeats.

Since live/dead assay using confocal microscopy showed that there were both live and dead bacteria after MMP1 treatment and did not show a consistent pattern (**Fig 4**), CFU assay was performed to quantify only the live cells (see Methods for detailed procedures). Moreover, we used catalytically inactive E219Q point mutant MMP1 to confirm that the observed activities on biofilms were indeed due to MMP1 activity. The E219Q mutation has been shown to inhibit MMP1 activity on collagen [39–42], the well-known substrate for MMP1. CFU assay showed that active MMP1 clearly inhibited biofilm growth up to 7 days for both strains (**Fig 5A**, green bars), as compared to control experiments with BHI media, protein buffer, and inactive MMP1 (**Fig 5B**, blue, magenta, and red bars respectively). However, disruption of 7-day-old biofilm was not significant.

### Congo red staining assay to quantify the overall integrity in inhibition and disruption of biofilms by MMP1

**Fig 6:**
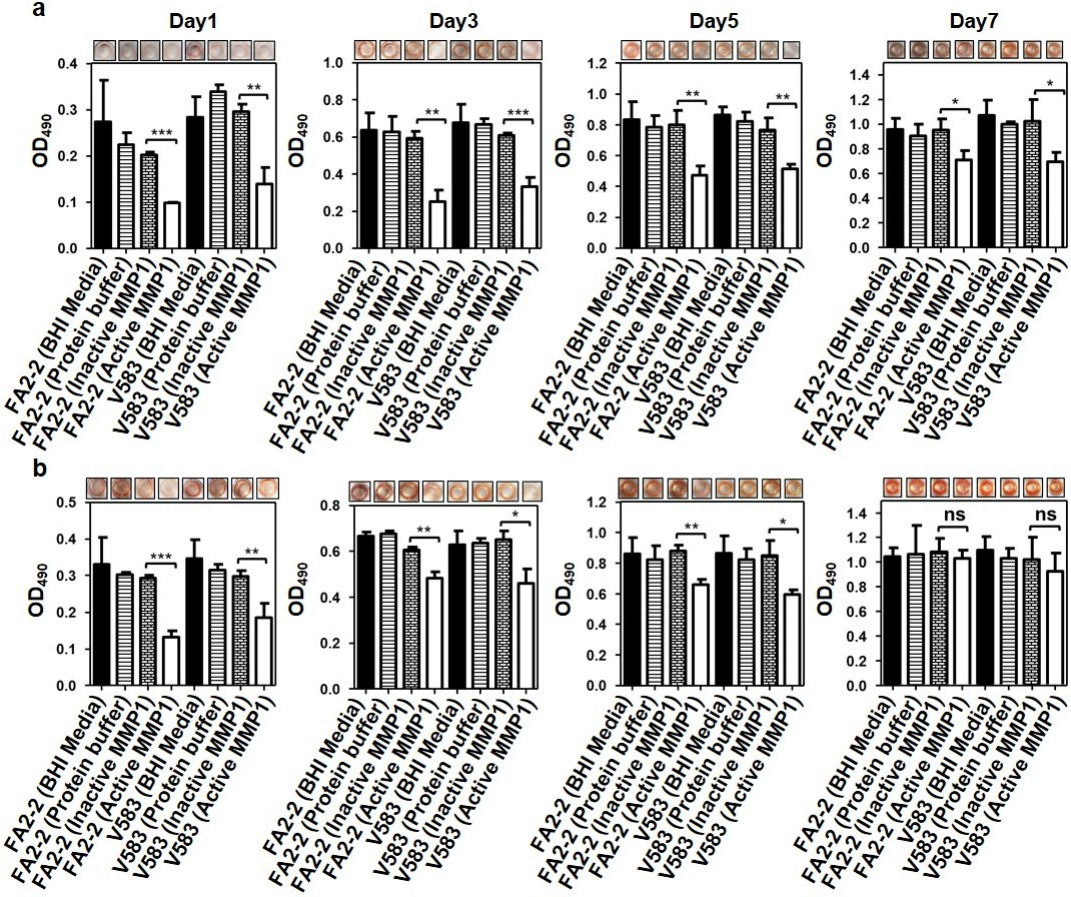
Congo red staining assay to quantify effects of MMP1 on *E*. *faecalis* biofilms. Absorption of Congo red stain at 490 nm (OD_490_) of 1, 3 and 7 day old *E*. *faecalis* FA2-2 and V583 biofilms treated with and without MMP1 under inhibition (**a**) and disruption (**b**) conditions Experiments with BHI media and protein buffer were used as controls. For inhibition experiments, biofilms were grown in presence of MMP1 from day0 to day7. For disruption experiments, biofilms were first grown in BHI media without MMP1 for desired duration, followed by MMP1 treatment. Typical images of wells in microtiter plates are given at the top of each panel. * indicates p-value: *p<0.01, **p<0.01 and ***p<0.001; ns indicates that the effect is not significant. Error bars on the data points represent the standard deviations of 3 technical repeats.

While CV assay, SEM, live/dead assay using confocal, and CFU assay showed that MMP1 affected biofilms of *E*. *faecalis*, the possible mechanisms were not clear. Since MMP1 is a protease, we postulated that MMP1 inhibits and disrupts the overall biofilm structure by degrading proteins in biofilms. To test this hypothesis, we performed Congo red assay [43, 44] (see Methods for detailed procedures). Since Congo red can stain both proteins and polysaccharides, we used Congo red staining assay to evaluate degradation of the overall biofilm structure by MMP1. As shown in **Fig 6**, active MMP1 degraded proteins in biofilms significantly, but catalytically inactive MMP1 showed no significant degradation.

### Absorption measurement to quantify *E. faecalis* planktonic growth with and without MMP1

**Fig 7:**
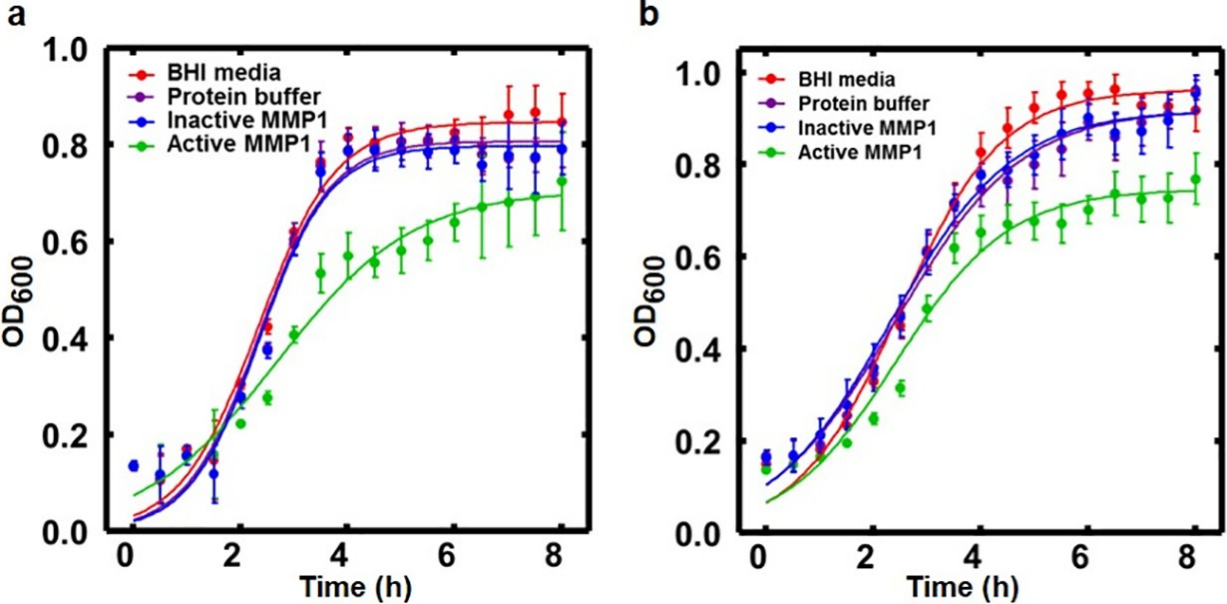
Effect of MMP1 on *E*. *faecalis* growth. Growth curves of *E*. *faecalis* in presence of active MMP1, BHI media, and protein buffer for **(a)** FA2-2 and **(b)** V583 strains respectively. Symbols represent data points. Solid lines are fits to the logistic equation of bacterial growth, *y* = *a*/{1+*b*exp(−*kt*)}, where *k* represents the growth rate. Error bars on the data points represent the standard deviations of 6 technical repeats. For FA2-2 strain, the best fit parameters are: *a* = 0.85±0.01, *b* = 24.98±4.44, *k* = 1.38±0.08 (BHI media); *a* = 0.81±0.01, *b* = 32.53±7.02, *k* = 1.49±0.09 (protein buffer); *a* = 0.80±0.01, *b* = 36.62±8.88, *k* = 1.53±0.10 (inactive MMP1); *a* = 0.71±0.02, *b* = 8.47±1.22, *k* = 0.79±0.06 (active MMP1). For V583 strain, the best fit parameters are: *a* = 0.96±0.01, *b* = 13.41±1.20, *k* = 1.05±0.04 (BHI media); *a* = 0.92±0.01, *b* = 7.70±0.62, *k* = 0.85±0.04 (protein buffer); *a* = 0.92±0.01, *b* = 7.73±0.65, *k* = 0.88±0.04 (inactive MMP1); *a* = 0.75±0.01, *b* = 10.12±1.21, *k* = 0.96±0.05 (MMP1). For fit parameters, error bars represent the standard error of the mean.

Inhibition and disruption of biofilms by MMP1 motivated us to evaluate the effect of MMP1 against cell growth. *E*. *faecalis* growth was observed for 8 hr in media supplemented with active MMP1, BHI media, and protein buffer. Absorption measured by the optical density at 600 nm (OD_600_) was significantly decreased in MMP1-treated samples after 2 hr compared to controls (**Fig 7A** and **7B**). Maximum difference was observed after 4–6 hr of incubation. Results clearly showed that MMP1 treatment significantly reduced the growth of *E*. *faecalis* cells.

## Discussion

### MMP1 inhibits and disrupts established biofilms of *E. faecalis*

Biofilms are multi-layered three dimensional macrocolonies of bacterial cells attached to biotic or abiotic surfaces encapsulated in an exopolysaccharide matrix [45]. Biofilm architecture and exopolysaccharide matrix play important roles in the formation of bacterial biofilms and their dispersal [4]. Thicker biofilm becomes impermeable to antimicrobial agents, and provides a favorable microenvironment for bacterial cells to grow [46]. For example, *Pseudomonas aeruginosa* forms multilayered biofilms with typical mushroom-shaped structure and these biofilms are highly resistant to detergent, antiseptics and antibiotics [47, 48]. *Staphylococcus aureus* biofilms alter matrix structure in order to convert from a multiplication stage to a dispersal stage for dissemination of planktonic bacteria [49]. Similarly, *E*. *faecalis* EPS contributes to augmented cell survival under energy-deficient conditions [50]. To study *E*. *faecalis* biofilms, CV staining and bright field microscopy is the most common method to quantify different stages of biofilm formation [51], inhibition, and disruption [34]. CV straining showed (**Figs 1** and **2**) that MMP1 inhibited and disrupted biofilms 2–3 times more compared to the controls. The effectiveness of MMP1 was significant against 1-day-old to 5-day-old biofilms; however, more mature 6-day-old or older biofilms exhibited resistance to the specific MMP1 dose used for 24 hr. The prominent effect on biofilms grown for less than 5 day is likely due to easier penetration of biofilms by MMP1. The mature biofilms older than 6 day were not disrupted effectively by MMP1 because of the protection by thick extracellular matrix layer [46], which agrees with the fact that *E*. *faecalis* biofilms can tolerate high concentrations of antimicrobial agents leading to emergence of antibiotic resistant phenotypes [52, 53].

### MMP1 reduces the planktonic growth rate of *E. faecalis*

Bacteria in suspension or planktonic culture differ from bacteria in biofilms [54]. Therefore, quantifying growth of planktonic cultures using absorption measurements offers a complementary view of the effects of MMP1. Absorbance or logarithm of the number of bacteria as a function of time showed a sigmoidal pattern and can be mathematically approximated by a logistic equation [55]. The solution of the logistic equation is, *y* = *a*/{1+*b*exp(−*kt*)}, where *a* is the asymptotic saturation value of the growth, *b* is the saturation value relative to the initial value, and *k* is the growth rate. **Fig 7** shows the planktonic growth profiles of FA2-2 and V583 strains of *E*. *faecalis*. MMP1 clearly reduced the growth rate of FA2-2 strains by more than 50%, but the reduction was less prominent for the V583 strain. Catalytically inactive mutant MMP1 did not reduce the growth rates indicating that the observed reduction in growth was due to catalytic activity of MMP1. One implication of correlation of MMP1 effect with vancomycin sensitivity is that MMP1 might disturb the development of the cell wall in growing bacteria similar to vancomycin [56]. The FA2-2 strain synthesizes cell-wall peptidoglycans ending in D-Ala-D-Ala, which binds vancomycin with high affinity and prevents cell wall synthesis. For the V583 strain, cell-wall peptidoglycan with different ending such as D-Ala-D-Lac is produced, which binds vancomycin with low affinity and does not prevent cell wall synthesis leading to bacteria growth. However, in the absence of vancomycin, both strains synthesize D-Ala-D-Ala. Therefore, the observed difference in the growth rates of FA2-2 and V583 is unlikely due to the interference of MMP1 on the development of cell-wall. Interestingly, the FA2-2 strain is gelatinase deficient due to the absence of a functional fsr system [57, 58]. Since MMP1 is a broad-spectrum protease and can act as a gelatinase [39], it is possible that MMP1 might reduce growth by penetrating cell wall and degrading intracellular proteins. Further studies are needed to determine these possibilities.

### MMP1 acts an antibiofilm agent by degrading proteins of *E. faecalis* and inhibiting growth

Even though the mechanism of antibiofilm effect is not completely clear, the broad-spectrum protease activity [39] of MMP1 provides some insights. MMP1 likely interferes with cell attachment leading to the spotty adherence and also breaks interconnected proteinaceous architecture of biofilms. MMP1 might also interfere with the function of membrane bound proteins leading to the growth inhibition of resident bacteria in biofilms. In fact, roles of proteases against biofilms have been studied extensively. Biofilm formation involves initial reversible attachment followed by irreversible attachment to the surface [8]. In the initial attachment stage, surface proteins and other antigens play essential roles and facilitate attachment [59]. Biofilm inhibition by an agent has been linked to degradation of surface and intracellular proteins in essential pathways that can reduce cell-to-cell or cell-to-surface adhesion [60] or interference with bacterial growth [61]. For example, *E*. *faecalis* surface protein (Esp) is known to facilitate surface attachment leading to biofilm formation. It has been shown that Esp insertion-deletion mutants form unstructured and weak biofilms [62]. An endogenous Streptococcal surface-protein-releasing enzyme (SPRE) has been shown to cause detachment of *Streptococcus mutans* biofilm via release of the surface protein antigen P1 [63]. Streptococcal Cysteine Protease (SpeB) has been found to be involved in hydrolysis of surface proteins M and F causing dispersal of *S*. *pyogenes* biofilms [64]. These proteins play key roles in cell attachment to biotic/abiotic surfaces and adjacent cells leading to biofilm formation. Degradation of surface proteins can affect bacterial physiology, cell growth, response to environmental stress [65], and transportation of large and hydrophobic molecules [66]. In this context, degradation of surface proteins by proteases is an effective strategy for biofilm inhibition and disruption. Proteases are known to degrade surface proteins and disrupt bacterial biofilms, degrade peptides in quorum sensing signaling pathways and disrupt intercellular communication [67]. Recently, aureolysin (Aur), a *staphylococcal* metalloprotease has been shown to degrade Bap and clumping factor b to disrupt *S*. *aureus* biofilms [68]. LapG protease produced by *Pseudomonas putida* modified outer membrane-associated, EPS-binding proteins and activated biofilm dispersal under starving conditions [69]. Proteolytic enzymes are also known to inhibit the growth of *Neisseria gonorrhoeae* by degradation of surface proteins [70]. Trypsin, chymotrypsin, and proteinase K are well-known to cause lysis and growth inhibition of *Streptococcus agalactiae* and *Streptococcus dysgalactiae* [61, 71]. Culture supernatant containing extracellular protease of *Actinomycetes* culture inhibits *Staphylococcus aureus* biofilm formation [72]. A secreted protease (PrtA) was also found to inhibit cell growth, biofilm formation and pathogenicity of the plant pathogen *Xylella fastidiosa* [73]. More recently, Esp protease from *S*. *epidermidis* controlled colonization and inhibited biofilm formation of *S*. *aureus* [60].

Therefore, the observed effect on biofilm due to MMP1 is consistent with the fact that proteases can disrupt biofilm architecture by degrading extracellular matrix proteins [19, 20] and may also enhance antimicrobial susceptibility of treated biofilms and decrease the effective biofilm disruption concentration of conventional antibiotics [74]. Both SEM (**Fig 3**) and confocal images (**Fig 4**) showed that MMP1-treated biofilms contained void spaces and appeared thinner as compared to the media and protein buffer controls. MMP1 seemed to affect the biofilms of FA2-2 more compared to the biofilms of V583. Two possible reasons are production of more biofilms for antibiotic resistant strains [75] and production of different biofilm components by *E*. *faecalis* strains [76]. As shown in **Fig 6**, Congo red assay clearly indicated that MMP1 significantly reduced the biofilm matrix proteins for both inhibition (MMP1 treatment during biofilm growth) and disruption (MMP1 treatment on established biofilms) experiments, which was absent in the case of inactive mutant MMP1. Indeed, MMPs, in particular MMP1, remodel the extracellular matrix [77] and catalyze a wide range of matrix and non-matrix substrates including gelatin, aggrecan, versican, casein, nidogen, serpins, tenasin-C, perlecan, IGFBP-2,3, α1-antichymotrypsin, α1-proteinase inhibitor, and pro-TNFα[78]. Since human MMP1 is a broad-spectrum protease [39], the antibiofilm effects might be observed on biofilms of other bacterial strains as well. Since bacterial proteins are essential for cell metabolism and survival, the observed degradation of extracellular proteins in biofilm matrix (**Fig 6**) suggests that MMP1 can potentially degrade intracellular proteins as well if MMP1 gets inside the bacterial cells, a possibility that requires further studies. Protease activity of MMP1 might also explain the observed reduction of CFU for both inhibition and disruption experiments (**Fig 5**), which indicates that MMP1 can act as both bacteriostatic and bactericidal agent. This observation is further supported by the reduced cellular growth observed in the presence of MMP1 (**Fig 7**), which is absent for inactive MMP1.

**Fig 8:**
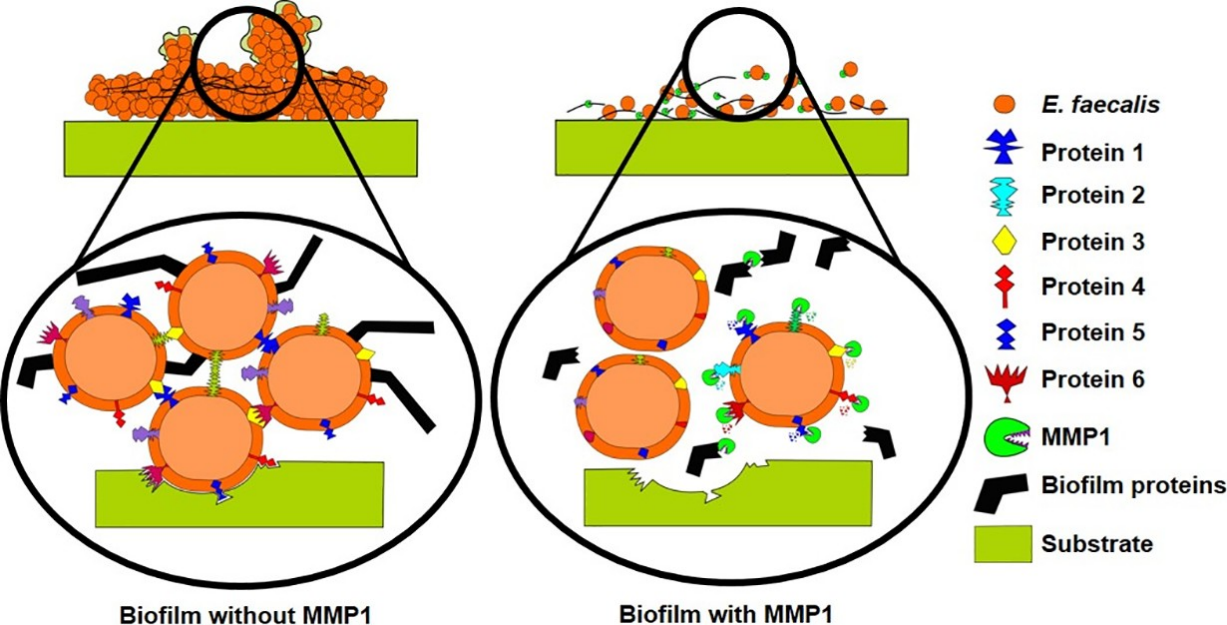
Mechanism of MMP1 antibiofilm activity against *E*. *faecalis*. MMP1 degrades cell-associated proteins and biofilm matrix components) leading to poor cell adhesion, bacterial growth inhibition, biofilm inhibition, and disruption (proteins 1–6 are hypothetical biofilm-associated proteins to illustrate the mechanism of MMP1 activity).

In conclusion, MMP1 showed potent antibiofilm activity against *E*. *faecalis* strains FA2-2 and V583. For inhibition experiments, biofilms did not grow well in the presence of MMP1 and any growth was easily aspirated with the solution during washing suggesting a loss of adherence. For disruption experiments, biofilms that were attached to the surface before treatment were either disappeared or became thinner due to reduced biofilm integrity and as such, were easily aspirated with the solution. While Congo red can stain both proteins and polysachcharides, the reduced biofilm mass due to MMP1 observed in the Congo red assay is potentially indicative of protein amyloids in *E*. *faecalis* biofilms. Indeed, amyloid fibers are now considered as common building block structures that confer stability to the biofilm matrix [79]. The likely degradation of amyloids by MMP1 has far reaching consequences in the context of other pathological amyloids. The MMP1 also reduced the planktonic growth of *E*. *faecalis* cells for both strains. Even though the antibiofilm effects and planktonic growth inhibition likely arise due to the protease activity of MMP1, further studies are need to define the exact mechanisms. As shown schematically in **Fig 8**, the antibiofilm activity of MMP1 is speculated to be associated with its broad-spectrum protease activity against bacterial proteins. While antibiofilm activity of proteases may not be surprising, the presence of MMP1 in the extracellular matrix and blood circulation potentially makes the antibiofilm activity of MMP1 an important aspect of defense mechanisms. These results also suggest that MMP1 may be used to formulate therapeutic strategies as broad-spectrum antibiofilm agent for the treatment of biofilm-associated infections.

## Materials and methods

### MMP1 purification

Active MMP1 was purified as described in a previous publication [39]. Briefly, the cDNA sequence of MMP1 was optimized for expression in *E*. *coli* and inserted into pET-21b (+) vector between NdeI (N-terminal) and HindIII (C-terminal) restriction sites. The plasmid was transformed into Rosetta (DE3) pLysS competent cells (Novagen, 70956). The cells were cultured in Luria Broth media (Sigma, L3022) to reach the optical density, OD_600_ = 0.1 (pH 7.0) at 37°C at 250 rpm in presence of chloramphenicol and ampicillin at the final concentrations of 34 μg/μl and 100 μg/μl respectively. The cells were induced for 5 hr with 1 mM Isopropyl β-D-1-thiogalactopyranoside (IPTG) and harvested after centrifugation. 1 g of the centrifuged cells was reconstituted in 7 ml of lysis buffer (pH 9.0) containing 50 mM Tris base (Sigma, T4661), 100 mM NaCl (Sigma, S9888), 200 μM ZnCl_2_ (Sigma, 208086), 400 μM CaCl_2_ (Sigma, 746495), freshly prepared 1% Triton X-100 (Sigma, T8787), 0.1 mg/ml trypsin (Worthington, TPCK-treated and irradiated, LS003750), and 1 mg/ml lysozyme (Sigma, L6878). The reconstituted cells were incubated for 18 hr at 37°C at 250 rpm and centrifuged to collect the supernatant, followed by centrifugation using 30 kD cut-off filters. MMP1 was quantified using Bradford assay and analyzed by SDS PAGE.

### Crystal violet staining to quantify inhibition and disruption effects

For inhibition experiments, biofilms were grown in the presence of MMP1. For disruption experiments, established biofilms were first grown in the absence of MMP1 and then treated with MMP1. Single colonies of *E*. *faecalis* cells were grown for 18 hr with 250 rpm shaking at 37°C in 5 ml of BHI media (BD, BBL Brain Heart Infusion Broth, 211059). To prepare log-phase cultures for inoculation, strains were subcultured in microtiter plates (Thermo Scientific, 96-well flat bottom sterile with lid and untreated, 266120) by adding 20 μl of overnight culture to 180 μl of BHI broth and incubated for 5 hr with 150 rpm shaking at 37°C to obtain OD_600_ ~0.5. For inhibition assays, 20 μl of OD ~0.5 bacterial inoculum was added to 50 μl of 1 mg/ml MMP1 and 180 μl of BHI media in each well. Until the day of quantifying inhibition using CV assay, the solution was aspirated from wells and fresh 50 μl of 1 mg/ml MMP1 and 200 μl of BHI media were added to the well every 24 hr. After the desired duration of incubation (1 to 7 days), the solution was aspirated from the well followed by washing with PBS buffer (Sigma, P3813, pH 7.4) to remove unbound planktonic cells, staining with 300 μl of 0.1% crystal violet stain (Sigma, C0775) for 15 min, washing three times with 300 μl of PBS buffer, dissolving CV stain in biofilm using 30% acetic acid (Sigma, A6283), and measuring absorption of CV stain dissolved in acetic acid at 635 nm using a plate reader (Biotek, Synergy2-Cam4, Software-Gen5-1.08). For disruption assay, 1-day-old to 7-day-old biofilms were grown in 96-well plates by adding 180 μl of BHI media and 20 μl of OD~0.5 bacterial inoculum in each well. After biofilms were established for the desired duration, we treated the biofilms with 50 μl of 1 mg/ml MMP1 for 24 hr at 37°C without shaking. MMP1-treated biofilms were then quantified using CV assay as described before. Effects of MMP1 were compared with two control experiments with BHI media and protein buffer containing 1% Triton X-100.

### Scanning electron microscopy of biofilm architecture

Established biofilms were first grown as follows. 300 μl of log-phase culture inoculation was added to 5 ml BHI media in wells of untreated 6-well plate (Celltreat Scientific Products, 229506), where biofilms were grown for 3 to 7 days by immersing 22 mm × 22 mm plastic coverslips (Carolina Biological Supply Company, 632900) in the solution. To study the effect of MMP1, 500 μl of 1 mg/ml MMP1 was added in each well. For control experiments, 500 μl of BHI media and protein buffer were added instead of MMP1. To image biofilms, the solution was aspirated and coverslips were washed three times with 500 μl of sterile PBS and dried at 37°C for 24 hr before imaging using a Phenom Pro-Scanning Electron microscope.

### Confocal laser scanning microscopy for live/dead assay

Biofilms were grown in the presence of MMP1 as described for SEM imaging. After brief air drying, biofilms were immediately stained with 500 μl of 10 μg/ml solution of propidium iodide (Thermo Fisher Scientific, Invitrogen, P3566) and 10 μg/ml acridine orange for 2 min. Coverslips were washed three times with 1 ml deionized water to remove unbound stain. Confocal laser scanning microscopic imaging was performed with a confocal microscope (Olympus, FV10i). Sensitivity was set at 40% for both the lasers. Images were processed using Fiji ImageJ software to merge green (live cells) and orange fluorescence (dead cells).

### Colony forming unit (CFU) assay to quantify live cells

Both inhibition and disruption effects were studied. To quantify live bacterial cells in biofilms, the standard plate count assay was performed. Biofilms (day 1 to day 7) were incubated with MMP1 and planktonic cells were washed with PBS buffer. After removing planktonic cells, cells inside biofilms were scrapped using a sterile tip and suspended in 100 μl of PBS buffer. A series of 10-fold dilutions were prepared and 100 μl of final dilutions were spread onto BHI plates using a sterile L-shaped spreader. CFUs were determined by counting the bacterial colonies on BHI plates. The total CFU counts were converted to Log_10_CFU and plotted.

### Congo red staining to quantify degradation of overall biofilm structure

Both inhibition and disruption effects were studied. After incubation of biofilms with MMP1, the solution was aspirated from wells. Biofilms were then washed with PBS buffer (Sigma, P3813, pH 7.4) to remove unbound planktonic cells. Staining was performed with 300 μl of 0.1% Congo red Hi Cert/ACS stain (Himedia, GRM508-10G) for 24 hr, followed by washing three times with 300 μl of PBS buffer. After washing, Congo red stain bound to biofilms was dissolved in DI water and absorption was measured at 490 nm using a plate reader.

### Absorption measurement to quantify *E. faecalis* growth

Log-phase cultures were grown as described before. 10 μl of log-phase culture was inoculated in 200 μl of sterile BHI broth in 96-well microtiter plates. 50 μlof 1 mg/ml MMP1 was added to obtain a total reaction volume of 260 μl. Absorption measurements at 600 nm were done every 30 min at 37°C using a plate reader. For control experiments BHI broth or protein buffer were used instead of MMP1.

### Statistical analysis

Data was analyzed using Graph Pad Prism 5.0 software. The effects of MMP1 treatment on biofilms were evaluated by Student's t-test. The effects were significant if the p-value less than 0.05.

## Supporting information

S1 Fig Visible inhibitory effects of MMP1 on *E*. *faecalis* FA2-2 biofilms on day 7. (**Top**) Wells after 7-day inhibition experiments. Coverslips are embedded within the solution and not visible. (**Bottom**) Wells after aspirating the solution. MMP1 shows clear inhibitory effect on biofilms.
(TIF)

S2 Fig SEM micrographs of established *E*. *faecalis* biofilms. Biofilms of vancomycin susceptible strain FA2-2 were first grown for 3 to 7 days and then treated with MMP1. In comparison to the control experiments, active MMP1 led to disruption of biofilms resulting in more empty spaces without any bacteria.
(TIF)

S3 Fig SEM micrographs of established *E*. *faecalis* biofilms. Biofilms of vancomycin susceptible strain V583 were first grown for 3 to 7 days and then treated with MMP1. In comparison to the control experiments, active MMP1 led to disruption of biofilms resulting in more empty spaces without any bacteria.
(TIF)
