# Supplementary figures and images for: Matrix metalloprotease-1 inhibits and disrupts Enterococcus faecalis biofilms
Source: PLoS One. 2019 Jan 11;14(1):e0210218. doi: 10.1371/journal.pone.0210218 (PMC6329490; doi:10.1371/journal.pone.0210218)

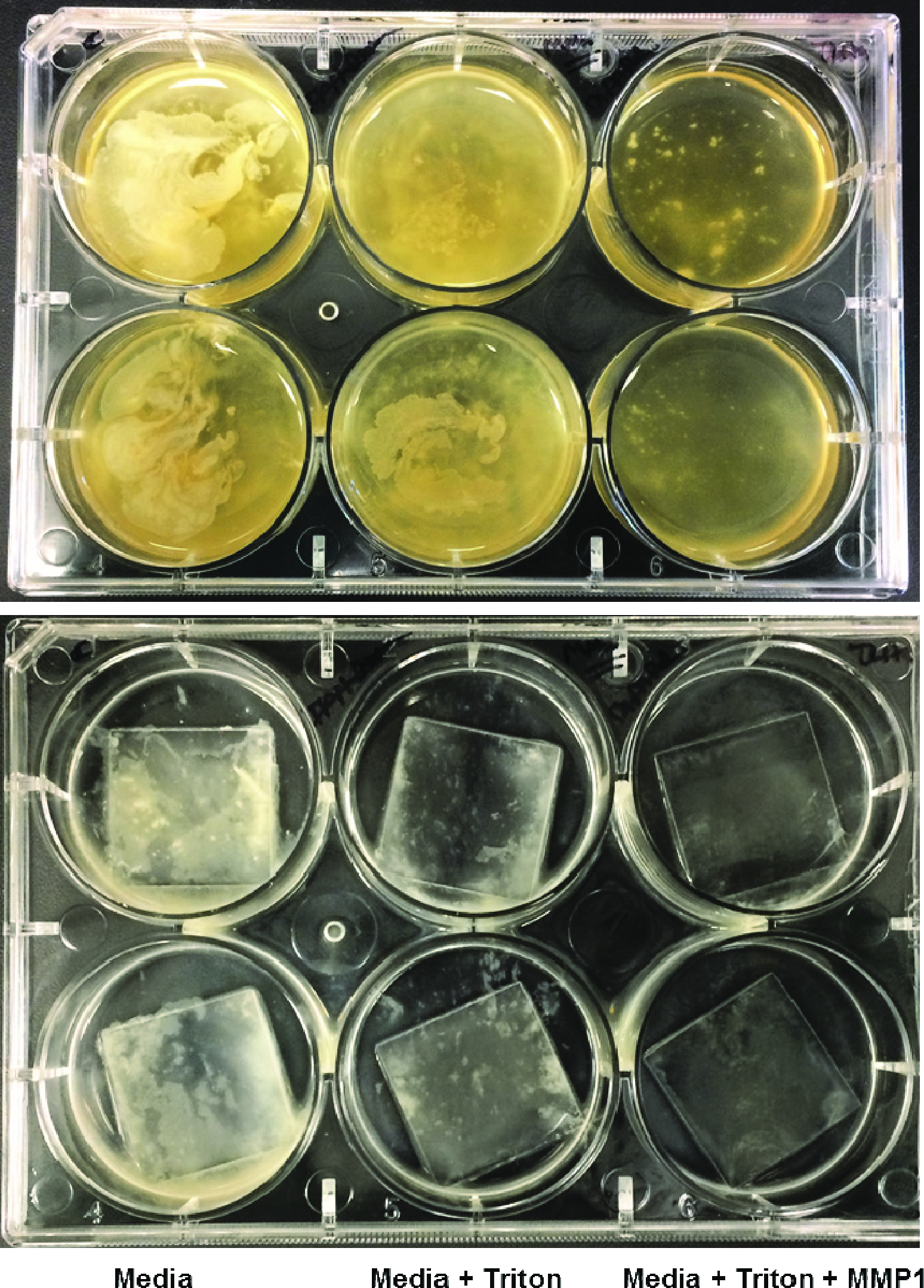

Supplement: S1 Fig — (Top) Wells after 7-day inhibition experiments. Coverslips are embedded within the solution and not visible. (Bottom) Wells after aspirating the solution. MMP1 shows clear inhibitory effect on biofilms. (TIF) [file pone.0210218.s001.tif]

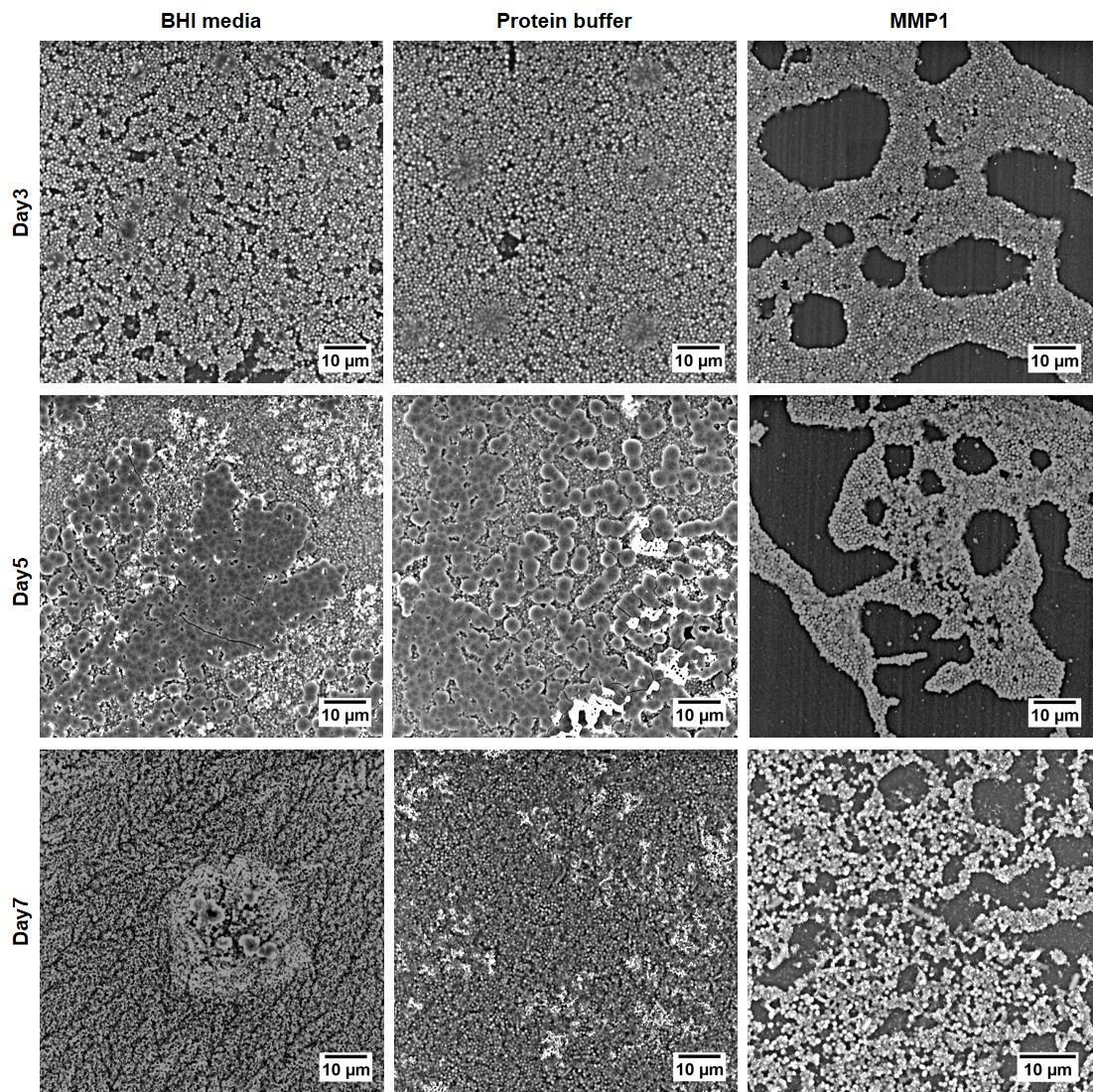

Supplement: S2 Fig — Biofilms of vancomycin susceptible strain FA2-2 were first grown for 3 to 7 days and then treated with MMP1. In comparison to the control experiments, active MMP1 led to disruption of biofilms resulting in more empty spaces without any bacteria. (TIF) [file pone.0210218.s002.tif]

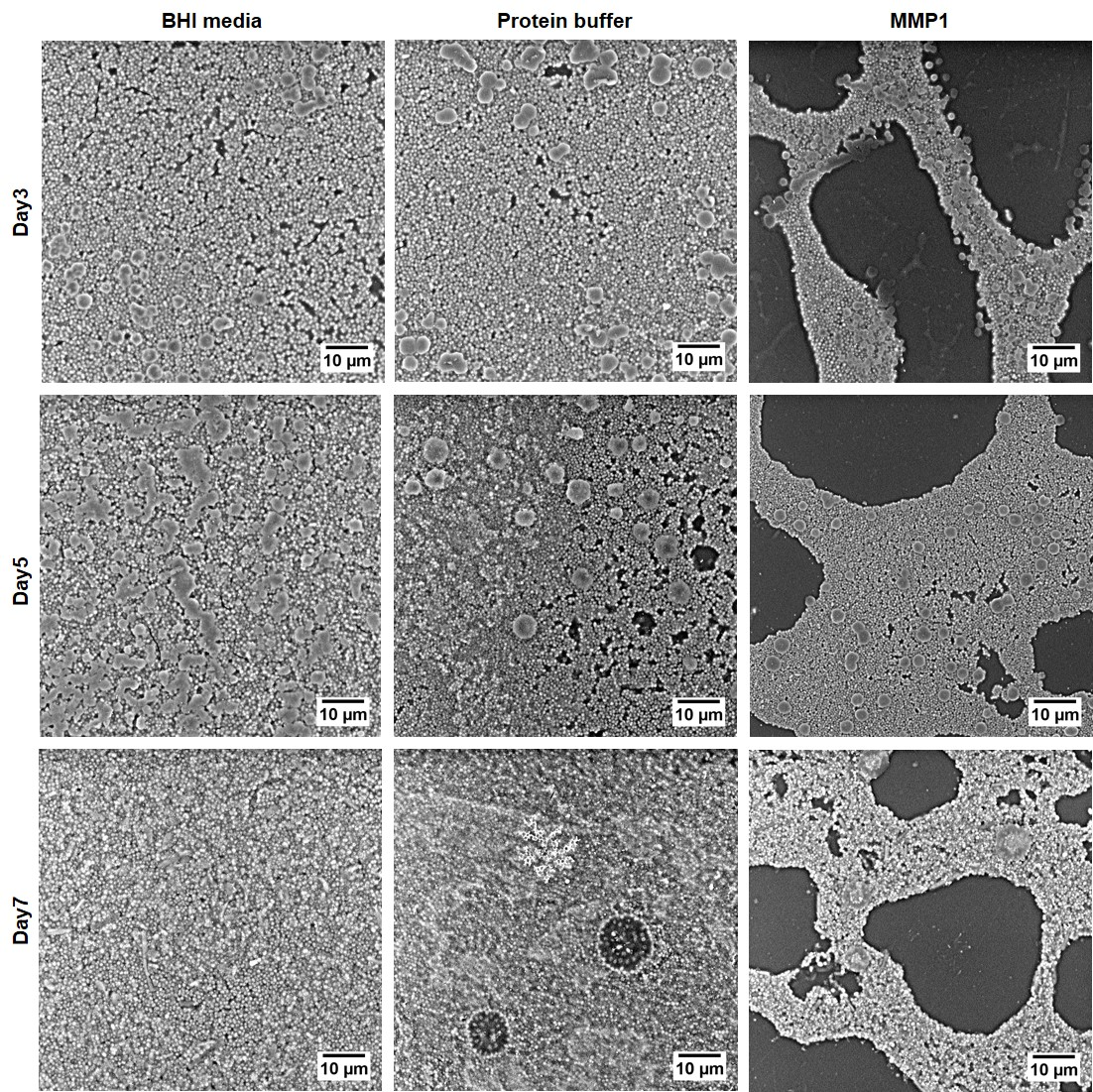

Supplement: S3 Fig — Biofilms of vancomycin susceptible strain V583 were first grown for 3 to 7 days and then treated with MMP1. In comparison to the control experiments, active MMP1 led to disruption of biofilms resulting in more empty spaces without any bacteria. (TIF) [file pone.0210218.s003.tif]
